# Supplementary material for: Ligand recognition and G protein coupling of the human itch receptor MRGPRX1
Source: Nat Commun. 2023 Aug 17;14:5004. doi: 10.1038/s41467-023-40705-z (PMC10435460; doi:10.1038/s41467-023-40705-z)
Supplement: Supplementary file 1 — Supplementary Information [file 41467_2023_40705_MOESM1_ESM.pdf]

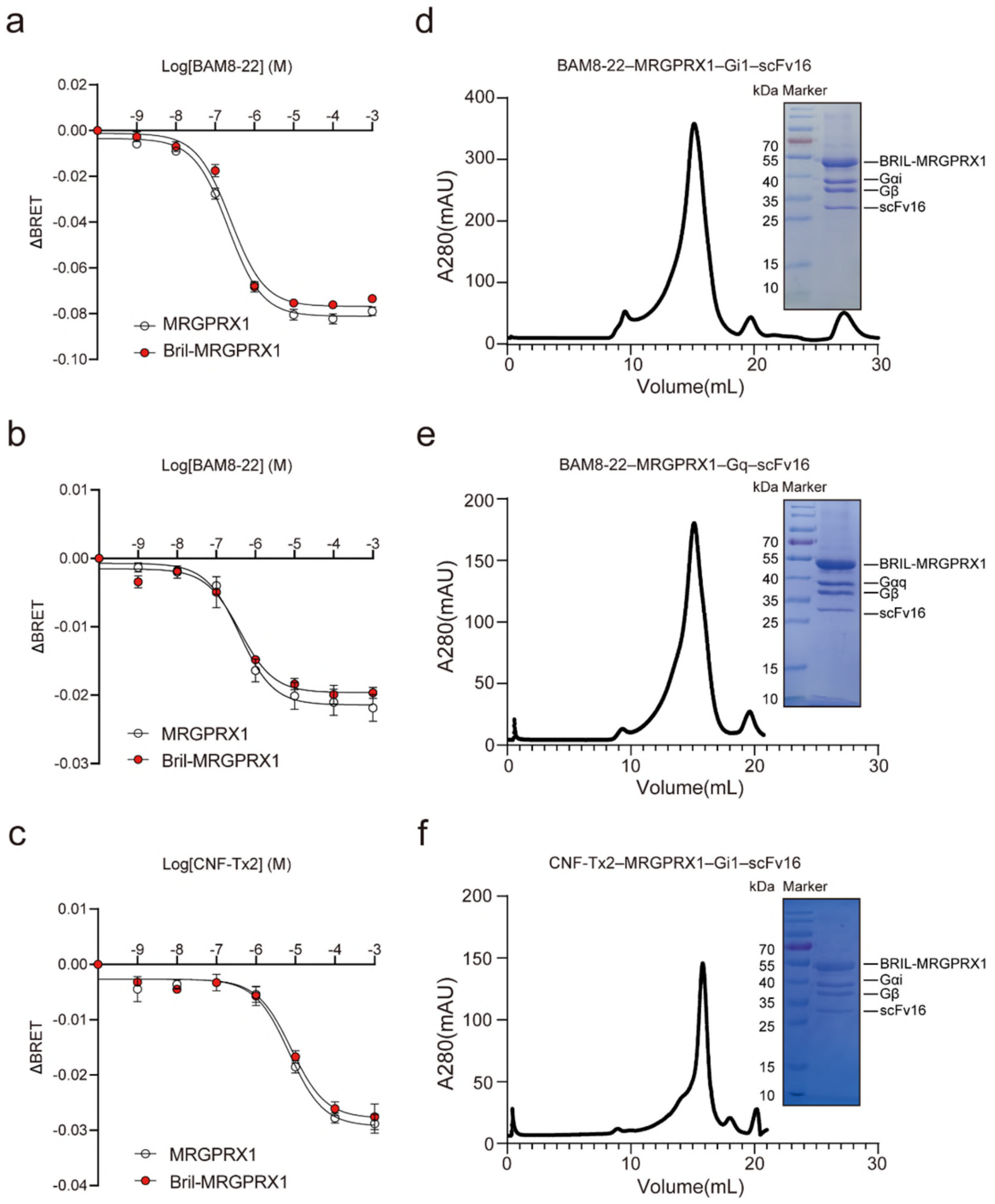

Supplementary Fig. 1 Purification of MRGPRX1-Gi1-scFv complex.

(a-b) Dose response curves of the BAM8-22 induced Gαi-Gγ dissociation (a) and Gαq-Gγ dissociation (b) in MRGPRX1-WT or Bril-MRGPRX1 overexpressing cells. Data from three independent experiments are presented as the mean ± SEM (n=3). **(c)** Dose response curves of the CNF-Tx2 induced Gαi-Gγ dissociation in MRGPRX1-WT or Bril-MRGPRX1 overexpressing cells. Data from three independent experiments are presented as the mean ± SEM (n=3). **(d)** Left panel: representative elution profile of BAM8-22-MRGPRX1-Gi1-scFv16 complex. BAM8-22-MRGPRX1-Gi1-scFv16 complex on Superose 6 Increase 10/300 column

and SDS-PAGE of the size-exclusion chromatography peak. Right panel, coomassie-stained PAGE of the isolated perk fraction from the Superose 6. **(e)** Left panel: representative elution profile of BAM8-22-MRGPRX1-Gq-scFv16 complex. BAM8-22-MRGPRX1-Gq-scFv16 complex on Superose 6 Increase 10/300 column and SDS-PAGE of the size-exclusion chromatography peak. Right panel, coomassie blue-stained PAGE of the isolated perk fraction from the Superose 6. **(f)** Left panel: representative elution profile of CNF-Tx2-MRGPRX1-Gi1-scFv16 complex. CNF-Tx2-MRGPRX1-Gi1-scFv16 complex on Superose 6 Increase 10/300 column and SDS-PAGE of the size-exclusion chromatography peak. Right panel, coomassie-blue stained PAGE of the isolated perk fraction from the Superose 6.

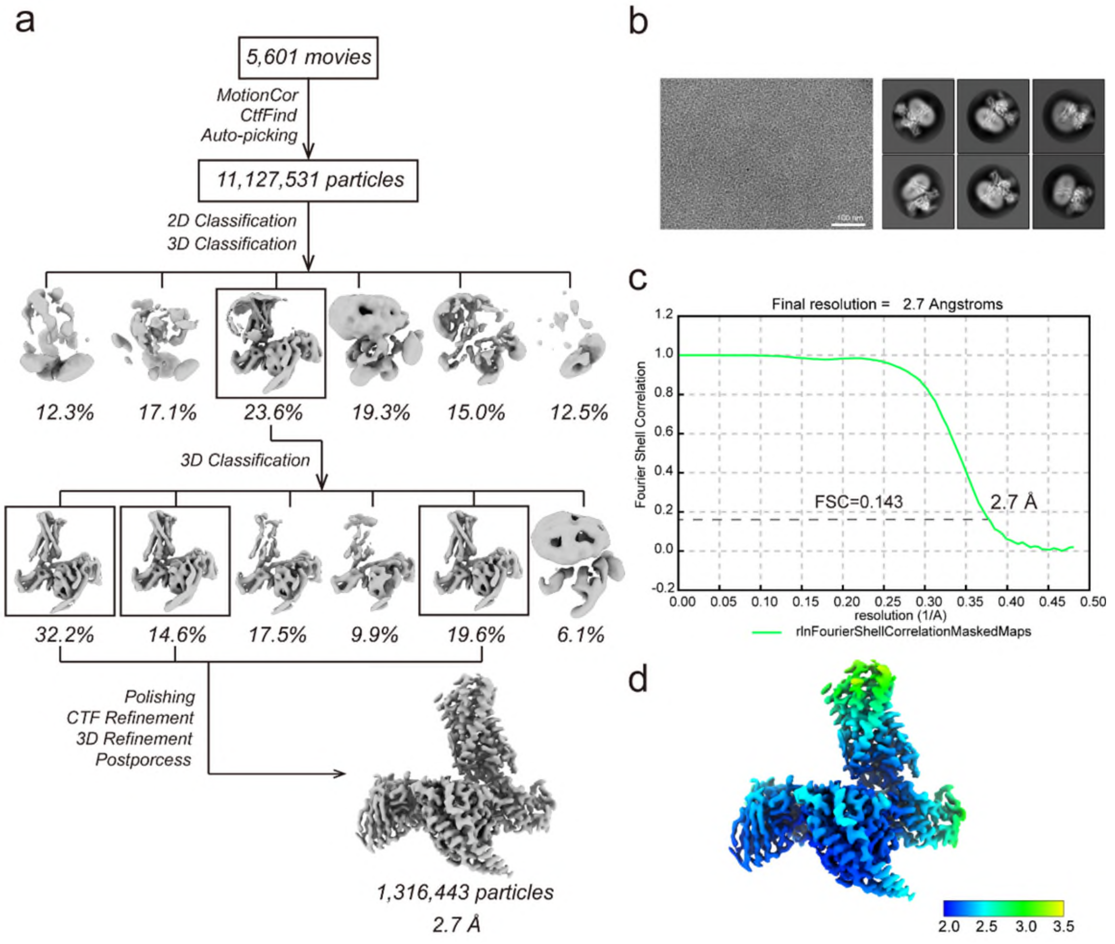

Supplementary Fig. 2 Cryo-EM images and single particle reconstruction of the BAM8-22-MRGPRX1-Gq complex.

(a) Flow chart for cryo-EM data processing of BAM8-22-MRGPRX1-Gq complex. **(b)** Representative Cryo-EM micrograph of BAM8-22-MRGPRX1-Gq complex (left) and 2D class averages (right). **(c)** Fourier shell correlation curves for the final 3D density maps of BAM8-22-bound MRGPRX1-Gq complex. At the fourier shell correlation (FSC) 0.143 cut-off, the overall resolution was 2.7Å. **(d)** 3D density map colored according to local resolution (Å) of the BAM8-22-MRGPRX1-Gq trimer complex.

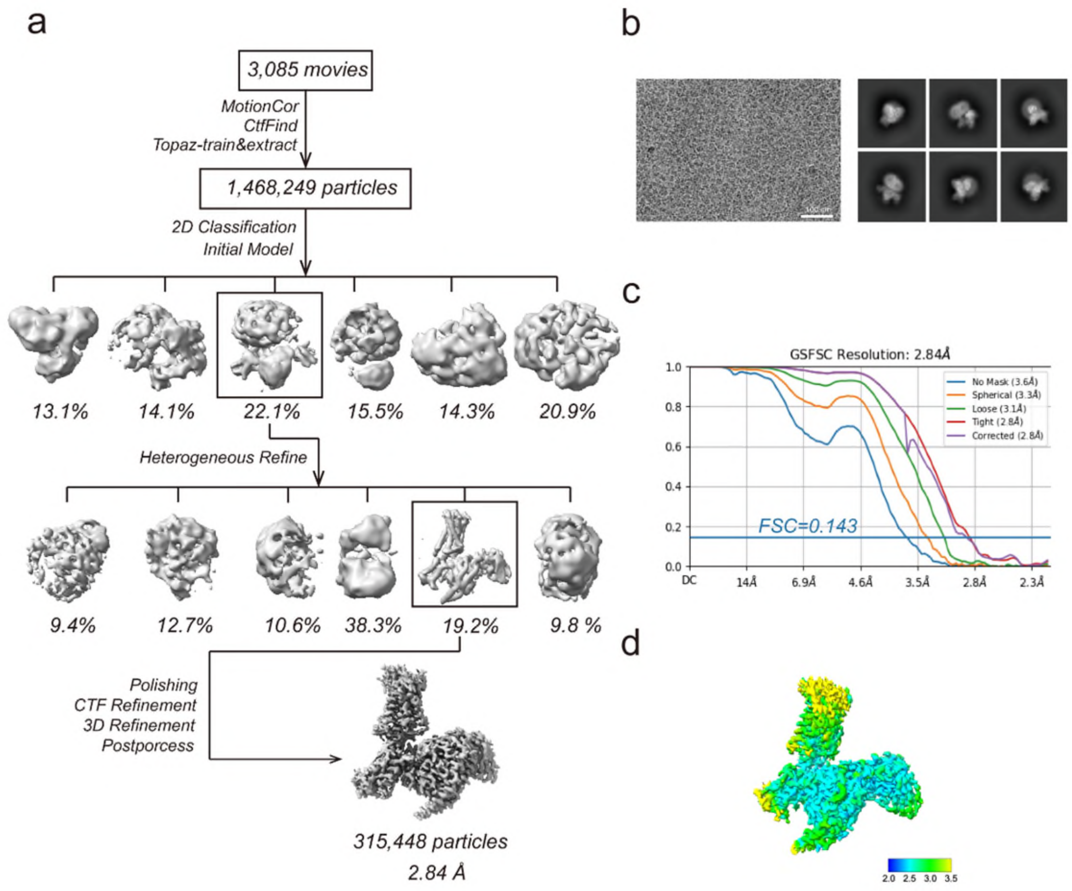

Supplementary Fig. 3 Cryo-EM images and single particle reconstruction of the CNF-Tx2-MRGPRX1-Gi1 complex.

(a) Flow chart for cryo-EM data processing of CNF-Tx2-MRGPRX1-Gi1 complex. **(b)** Representative Cryo-EM micrograph of CNF-Tx2-MRGPRX1-Gi1 complex (left) and 2D class averages (right). **(c)** Fourier shell correlation curves for the final 3D density maps of CNF-Tx2-MRGPRX1-Gi1 complex. At the fourier shell correlation (FSC) 0.143 cut-off, the overall resolution was 2.8 Å. **(d)** 3D density map colored according to local resolution (Å) of the CNF-Tx2-MRGPRX1-Gi1 trimer complex.

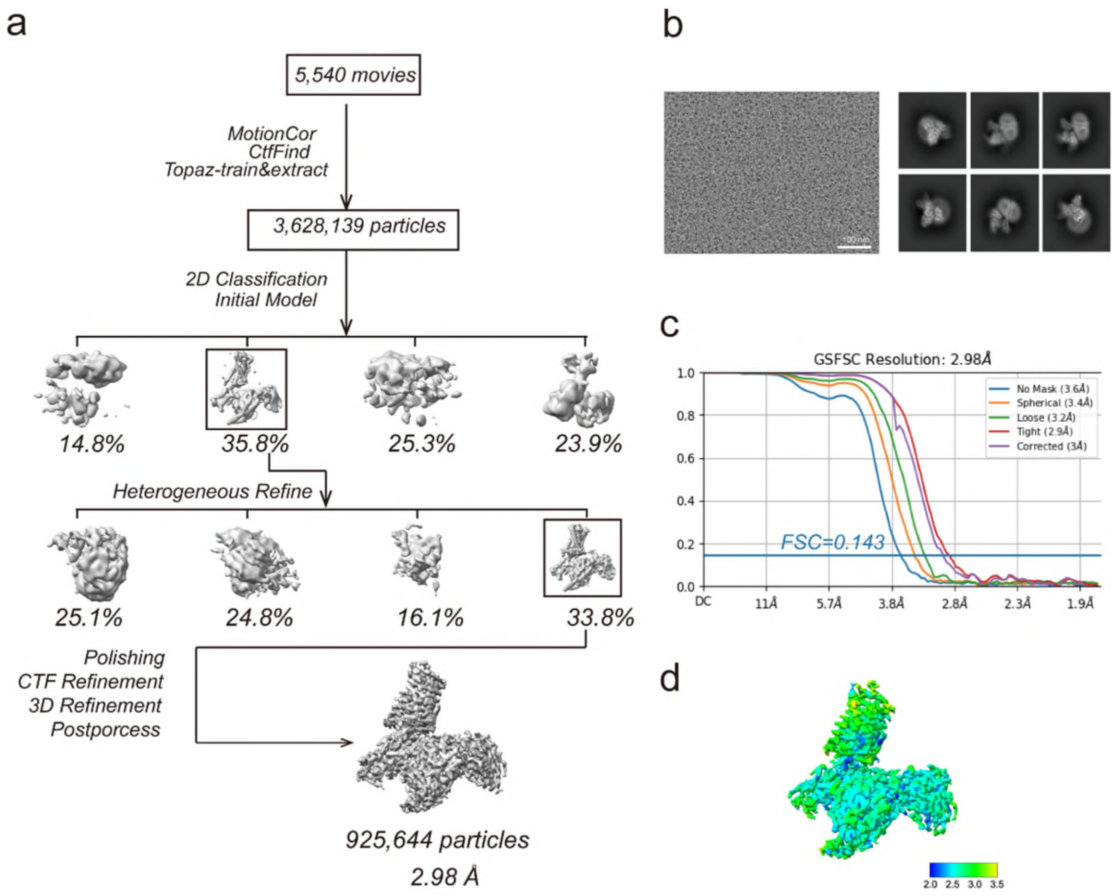

Supplementary Fig. 4 Cryo-EM images and single particle reconstruction of the BAM8-22-MRGPRX1-Gi1 complex.

(a) Flow chart for cryo-EM data processing of BAM8-22-MRGPRX1-Gi1 complex. **(b)** Representative Cryo-EM micrograph of BAM8-22-MRGPRX1-Gi1 (left) and 2D class averages (right). **(c)** Fourier shell correlation curves for the final 3D density maps of BAM8-22-bound MRGPRX1-Gi1 complex. At the fourier shell correlation (FSC) 0.143 cut-off, the overall resolution was 3.0 Å. **(d)** 3D density map colored according to local resolution (Å) of the BAM8-22-MRGPRX1-Gi1 trimer complex.

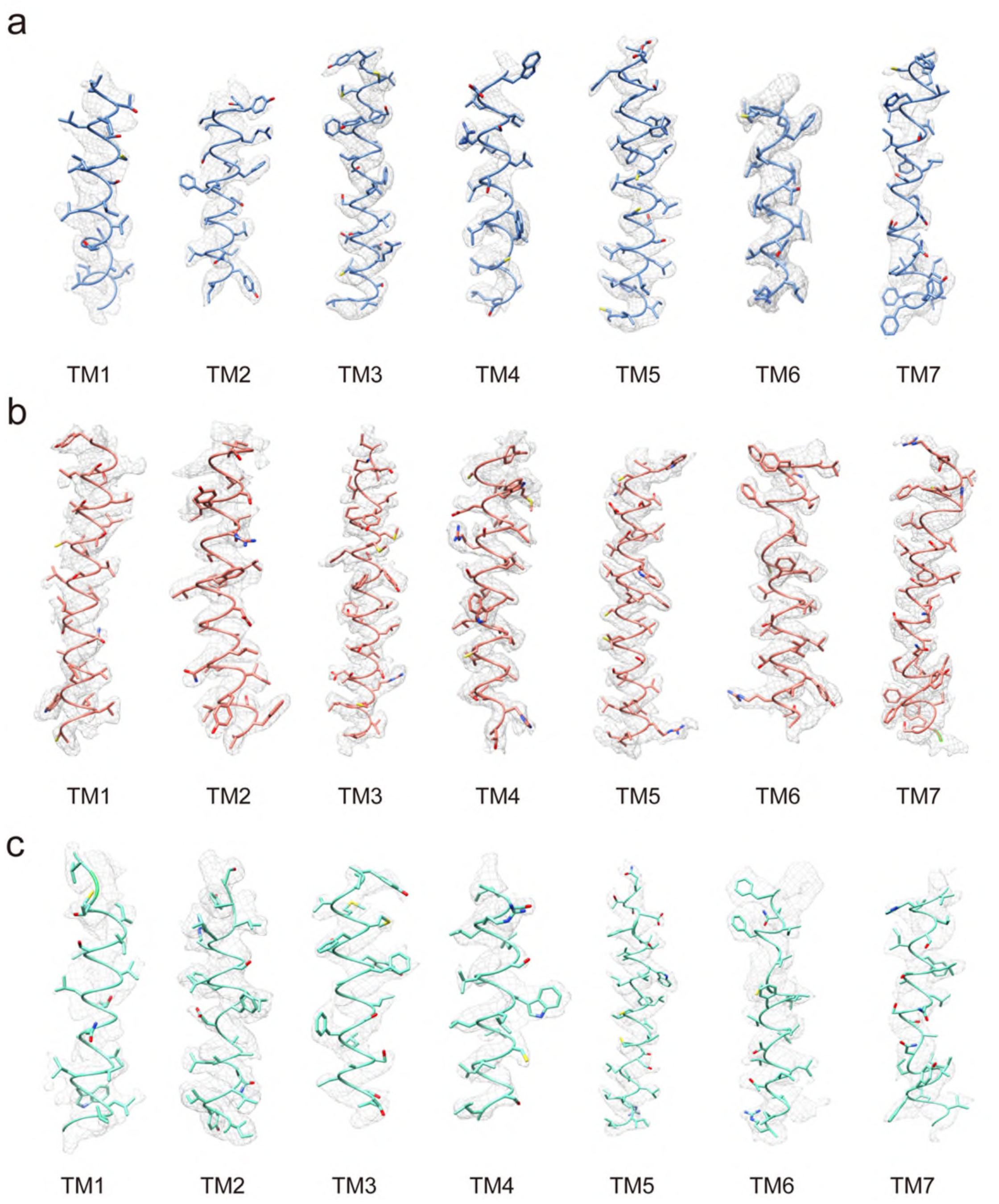

Supplementary Fig. 5 Electron microscopy density map of BAM8-22-MRGPRX1-Gi1, BAM8-22-MRGPRX1-Gq and CNF-Tx2-MRGPRX1-Gi1 complex.

Cryo-EM density of the transmembrane helices of MRGPRX1, including BAM8-22-MRGPRX1-Gi1 (**a**), BAM8-22-MRGPRX1-Gq (**b**) and CNF-Tx2-MRGPRX1-Gi1(**c**) cryo-EM structure respectively. All seven-TM bundles were unambiguously traceable in the cryo-EM density map, and the densities of large hydrophobic residues were utilized to assign the primary sequence of MRGPRX1.

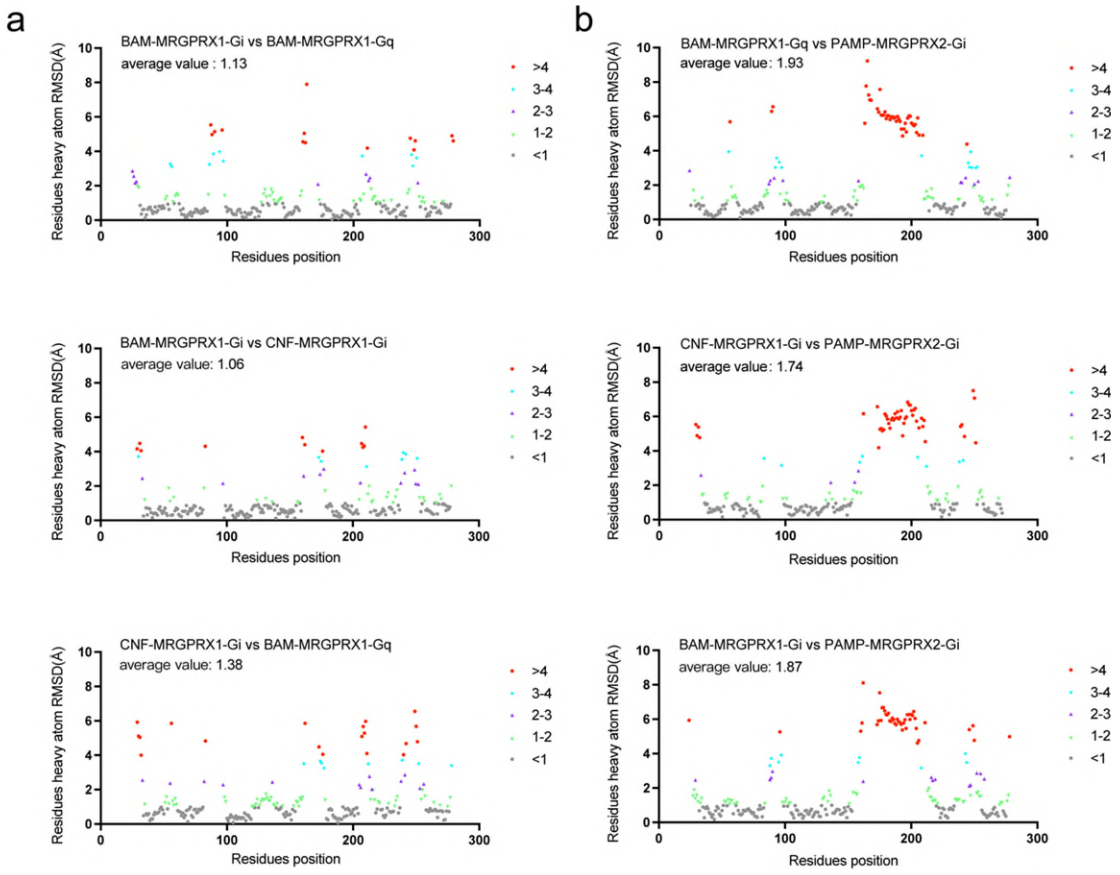

Supplementary Fig. 6 The root-mean-square-deviation (RMSD) of MRGPRX1 bound with different ligands or compared with MRGPRX2 structure.

(a) The root-mean-square-deviation (RMSD) of the overall structures of MRGPRX1 bound with different ligands assumed similar structures, including BAM8-22-MRGPRX1-Gi and BAM8-22-MRGPRX1-Gq (upper panel), BAM8-22-MRGPRX1-Gi and CNF-Tx2-MRGPRX1-Gi (middle panel), BAM8-22-MRGPRX1-Gq and CNF-Tx2-MRGPRX1-Gi (lower panel). **(b)** The root-mean-square-deviation (RMSD) of the overall structures of MRGPRX1, compared with our recently solved MRGPRX2 structure, including PAMP-12-MRGPRX2-Gi and BAM8-22-MRGPRX1-Gq (upper panel), PAMP-12-MRGPRX2-Gi and CNF-Tx2-MRGPRX1-Gi (middle panel), PAMP-12-MRGPRX2-Gi and BAM8-22-MRGPRX1-Gi (lower panel).

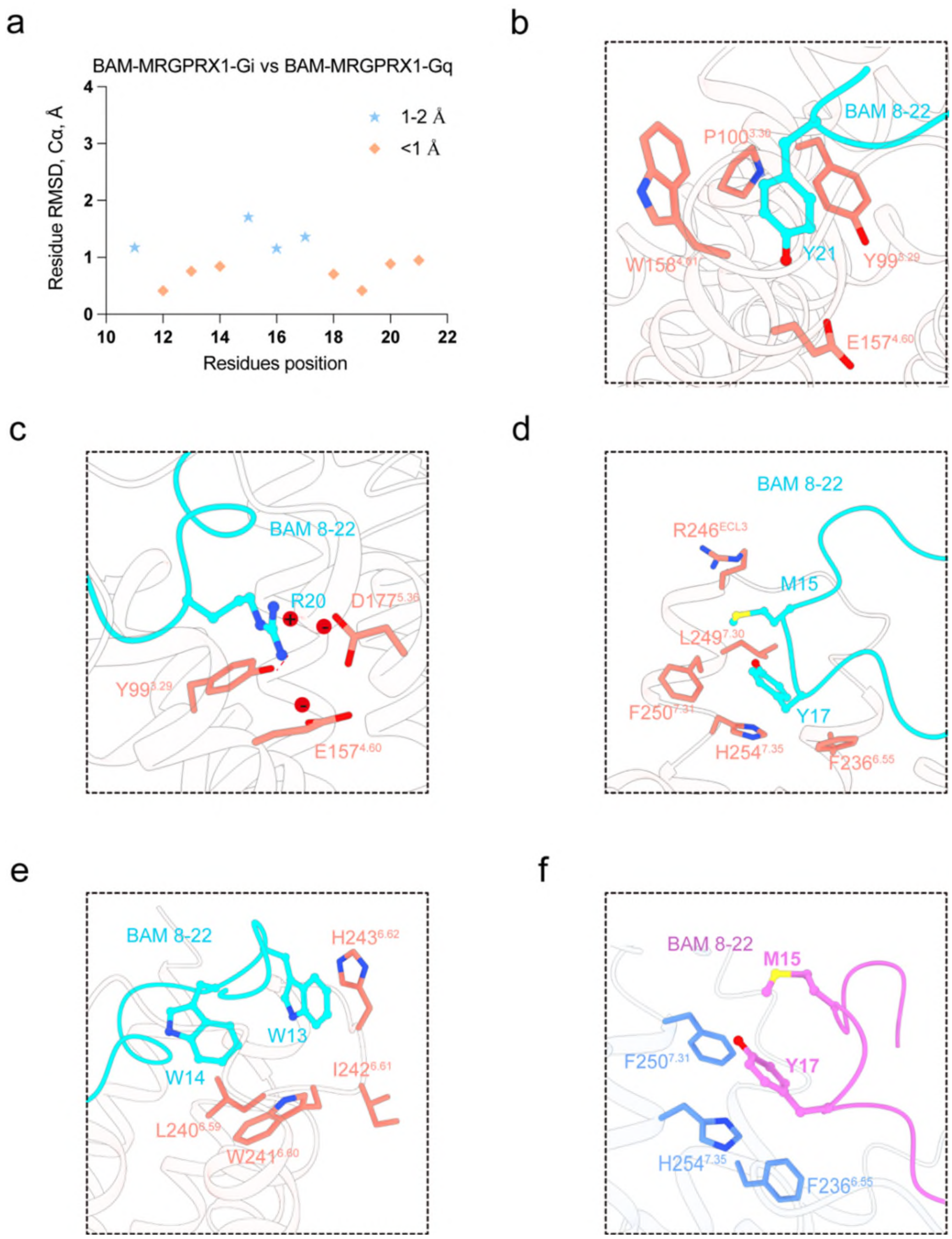

Supplementary Fig. 7 Binding of BAM8-22 to MRGPRX1.

(a) The root-mean-square-deviation (RMSD) of BAM8-22 between BAM8-22-MRGPRX1-Gi and BAM8-22-MRGPRX1-Gq complex structures. **(b-e)** Structural representations of the interactions surrounding the Y21(b), R20(c), M15 and Y17(d), W13 and W14(e) of MRGPRX1-Gq complex structures. Hydrogen bonds were shown in the red dash. **(f)** In the BAM8-22-MRGPRX1-Gi1 complex structure, the conformational changes of M15 and Y17 eliminated their interactions with R246^{ECL3} and L249$^{7.30}$.

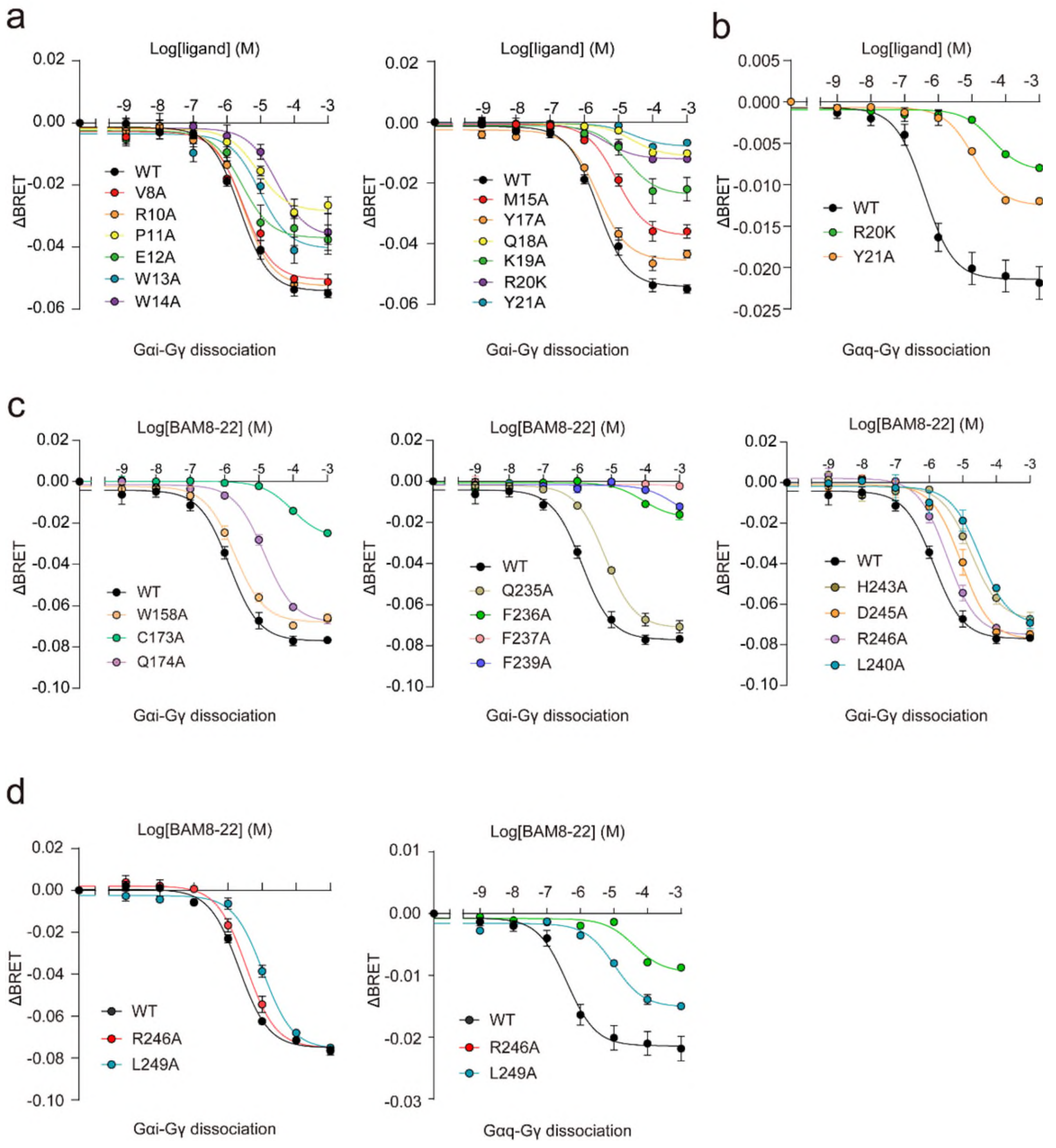

Supplementary Fig. 8 Effects of different mutations in BAM8-22 or different mutations within the ligand-binding pocket of MRGPRX1 induced Gαi-Gγ and Gαq-Gγ dissociation. **(a)** Effects of different BAM8-22 mutations on BAM8-22 induced Gαi-Gγ dissociation in MRGPRX1 overexpressing HEK293 cells. The curve data from three independent measurements are measured as mean ± SEM (n=3). **(b)** Effects of different BAM8-22 mutations on BAM8-22 induced Gαq-Gγ dissociation in MRGPRX1 overexpressing HEK293 cells. The curve data from three independent measurements are measured as mean ± SEM (n=3). **(c)** Effects of different mutations within the ligand-binding pocket of MRGPRX1 on BAM8-22 induced Gαi-Gγ dissociation in MRGPRX1 overexpressing cells. The curve data from at least three independent measurements are measured as mean ± SEM (n=3). **(d)** Effects of different mutations within the ligand-binding pocket of MRGPRX1 on BAM8-22 induced Gαi-Gγ

dissociation and Gαq-Gγ dissociation in MRGPRX1 overexpressing cells. The curve data from three independent measurements are measured as mean ± SEM (n=3).

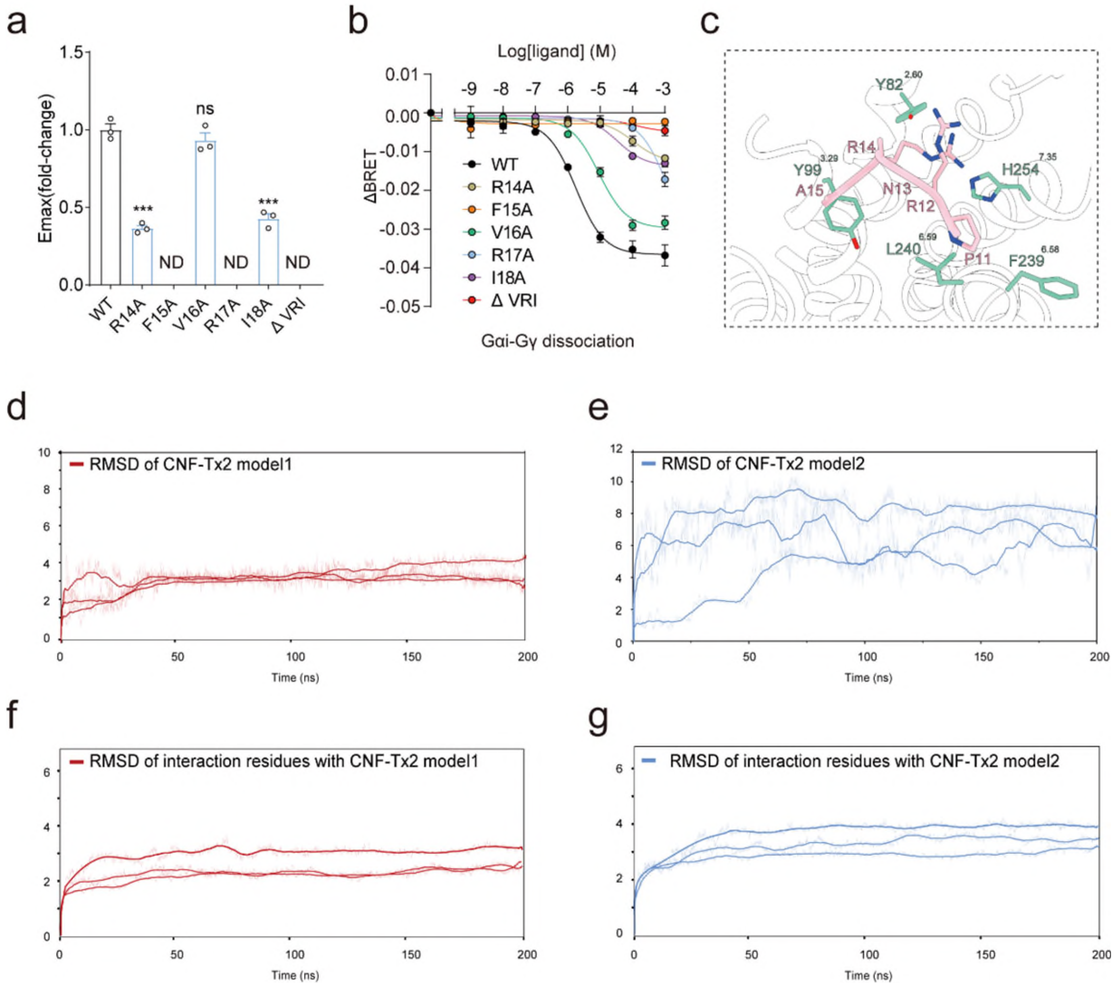

Supplementary Fig. 9 Binding of CNF-Tx2 to MRGPRX1.

(a) Emax effects of different mutations on CNF-Tx2 of MRGPRX1 induced Gαi-Gγ dissociation. The maximal response (Emax) is presented as the mean ± SEM of three independent experiments. Statistical differences between MRGPRX1 WT and mutations were determined by two-sided one-way ANOVA with Tukey test. *, P<0.05; **, P<0.01; ***, P<0.001; ns, no significant difference; ND, not detected. (P= P<0.001, ND, 0.6262, 0.109, P<0.001 from left to right). **(b)** Effects of different CNF-Tx2 mutations on CNF-Tx2 induced Gαi-Gγ dissociation in MRGPRX1 overexpressing HEK293 cells. The curve data from three independent measurements are measured as mean ± SEM (n=3). **(c)** Three-dimensional (3D) representation of the detailed interactions between CNF-Tx2 and MRGPRX1 in CNF-Tx2-MRGPRX1-Gi1-model2 complex. **(d-e)** The average RMSD value of CNF-Tx2 model1 (red) and CNF-Tx2 model2 (blue) during triplicate 200 ns MD simulations. **(f-g)** RMSD of key residues in MRGPRX1 which directly interact with CNF-Tx2 model1 (red) and CNF-Tx2 model2 (blue) during triplicate 200 ns MD simulations.

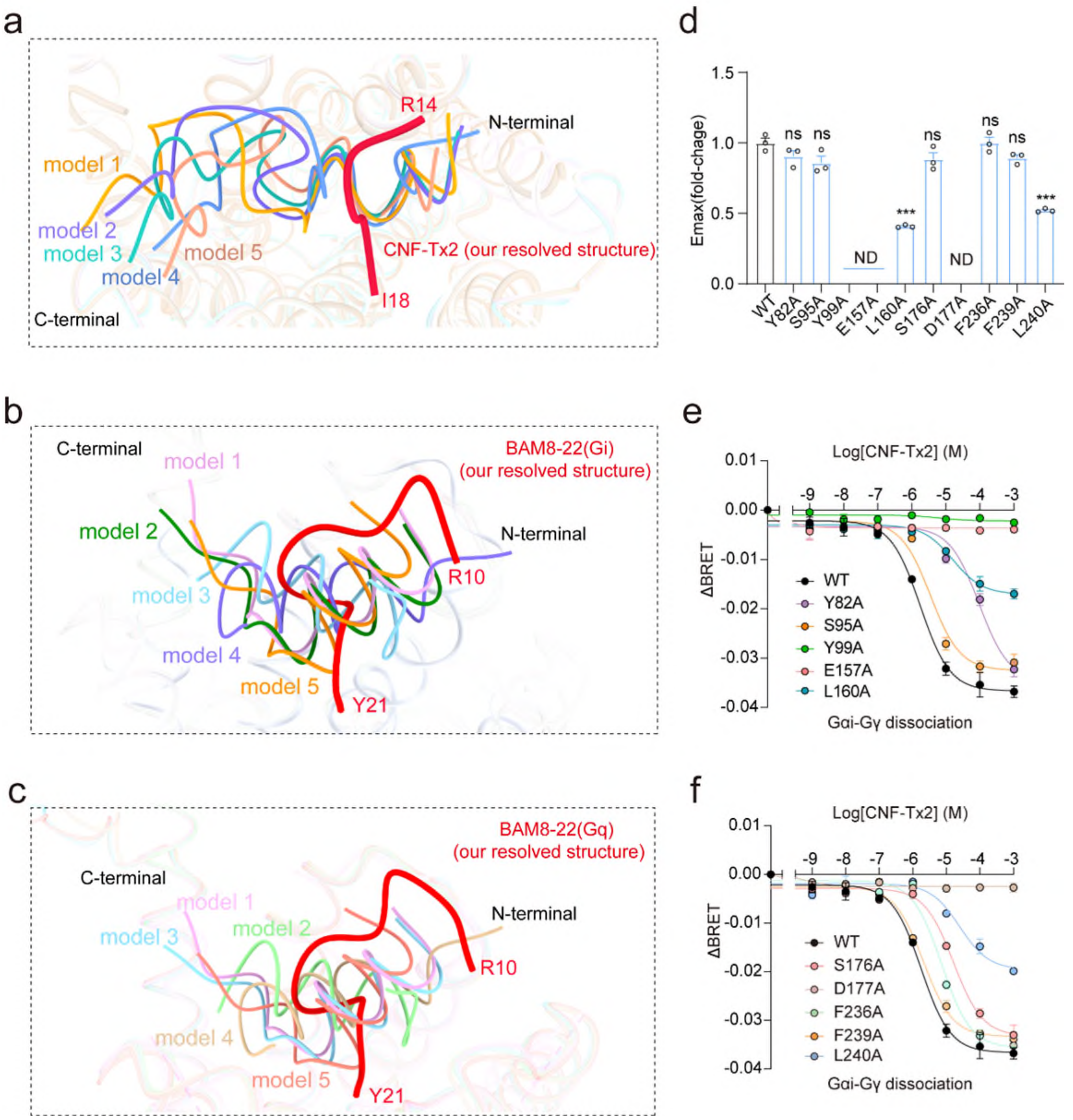

Supplementary Fig. 10 Binding of CNF-Tx2 and BAM8-22 to MRGPRX1.

(a) Comparison of CNF-Tx2 binding modes simulated by Colabfold and in CNF-Tx2-MRGPRX1-Gi1 complex structure that our resolved. The CNF-Tx2 model 1-5 predicted by Colabfold are shown in tangold, skybluemedium slate blue, plummedium turquoise, cornflower bluelightgreen and dark salmonwheat, CNF-Tx2 in our resolved CNF-Tx2-MRGPRX1-Gi1 complex is shown in red. **(b)** Comparison of BAM8-22 binding modes simulated by Colabfold and in BAM8-22-MRGPRX1-Gi1 complex structure that our resolved. The BAM8-22 model 1-5 predicted by Colabfold are shown in plum, dark green, light sky blue, medium purple and dark orange, BAM8-22 in our resolved BAM8-22-MRGPRX1-Gi1 complex is shown in red. **(c)** Comparison of BAM8-22 binding modes simulated by Colabfold and in BAM8-22-MRGPRX1-Gq complex structure that our resolved. The BAM8-22 model 1-5 predicted by Colabfold are shown in plum, light cyan, light sky blue, tan and light coral, BAM8-22 in our

resolved BAM8-22-MRGPRX1-Gq complex is shown in red. **(d)** Emax effects of different mutations within the ligand-binding pocket of MRGPRX1 on CNF-Tx2 induced Gαi-Gγ dissociation. Statistical differences between MRGPRX1 WT and mutations were presented as the mean ± SEM of three independent experiments and determined by two-sided one-way ANOVA with Tukey test. *, $P<0.05$; **, $P<0.01$; ***, $P<0.001$; ns, no significant difference; ND, not detected. (P=ns, ns, ND, ND, <0.001, ns, ND, ns, ns, <0.001 from left to right). **(e-f)** Effects of different mutations within the ligand-binding pocket of MRGPRX1 on CNF-Tx2 induced Gαi-Gγ dissociation in MRGPRX1 overexpressing cells. The curve data from three independent measurements are measured as mean ± SEM (n=3).

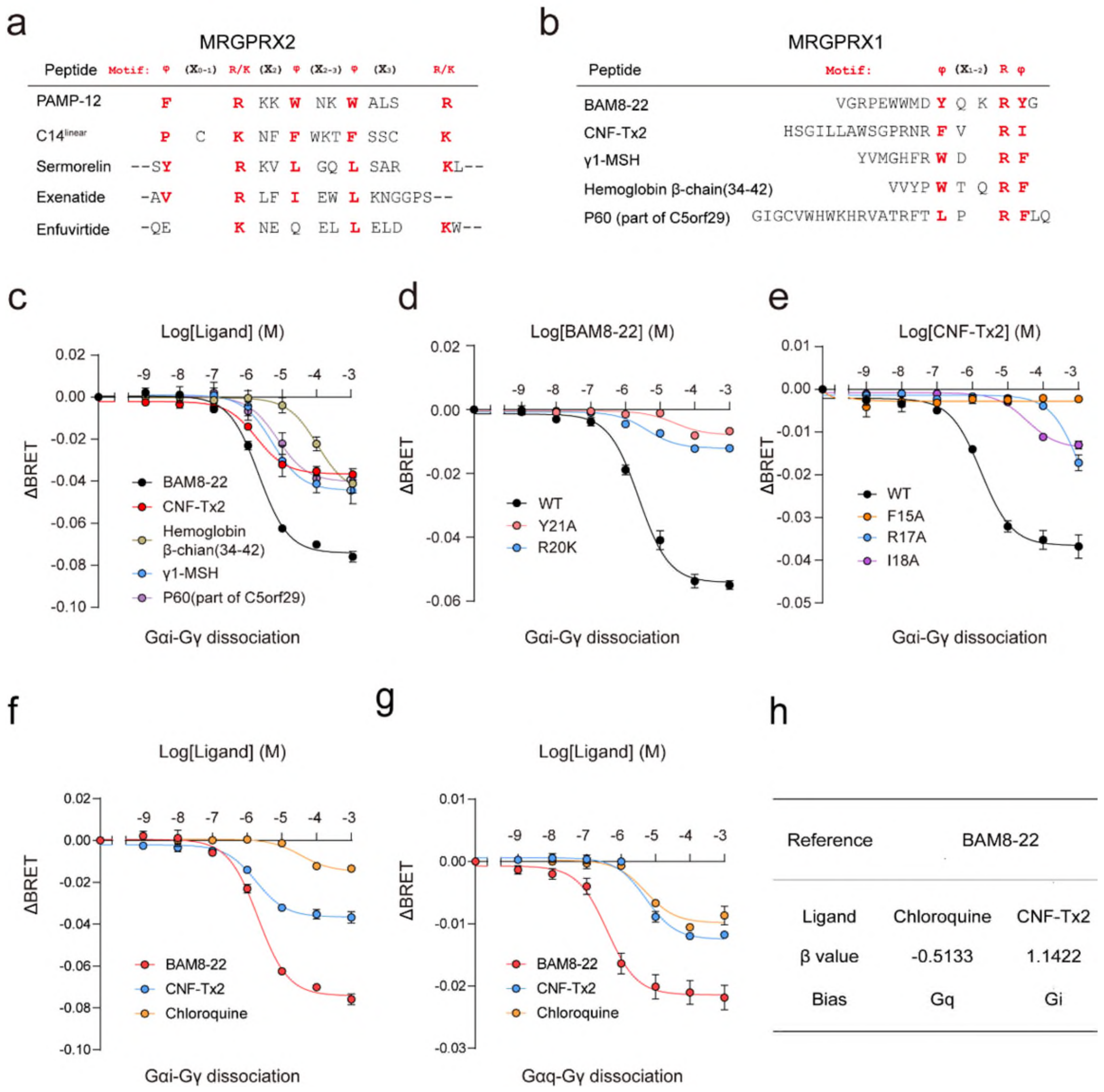

Supplementary Fig. 11 Sequence alignment of peptide common motif recognized by MRGPRX1 and MRGPRX2.

(a) Peptide ligand sequences of MRGPRX2. Sequence comparisons of several peptide-based allergens with similarities to the $\varphi^{P9}(X_{0\text{-}1})$ R/K$^{P10}(X_2)$ $\varphi^{P13}(X_{2\text{-}3})$ $\varphi^{P16}(X_3)$ R/K^{P20} motif. **(b)** Peptide ligand sequences of MRGPRX1. Sequence comparisons of several peptide-based allergens with similarities to the $\varphi^{B17}(X_{1\text{-}2})$ R^{B20} φ^{B21} motif. **(c)** Effects of BAM8-22, CNF-Tx2, γ1-MSH, hemoglogbin β-chain, P60 (part of C5orf29) induced MRGPRX1 activation evaluated via Gαi-Gγ dissociation assay. Data from three independent experiments are presented as the mean ± SEM (n=3). All data were analyzed by two-sided one-way ANOVA with Turkey test. **(d)** Effects of different BAM8-22 mutations on BAM8-22 induced Gαi-Gγ dissociation. Data from three independent experiments are presented as the mean ± SEM (n=3). All data were analyzed by two-sided one-way ANOVA with Turkey test. **(e)** Effects of different CNF-Tx2 mutations on CNF-Tx2 induced Gαi-Gγ dissociation. Data from three independent experiments

are presented as the mean ± SEM (n=3). All data were analyzed by two-sided one-way ANOVA with Turkey test. **(f)** Concentration-dependent response curves of MRGPRX1 in response to ligands by Gαi-Gγ dissociation assay. Values are mean ± SEM from three independent experiments (n=3) performed in triplicates. **(g)** Concentration-dependent response curves of MRGPRX1 in response to ligands by Gαq-Gγ dissociation assay. Values are mean ± SEM from three independent experiments (n=3) performed in triplicates. **(h)** Comparison of the biased properties of CNF-Tx2 and Chloroquine. Both CNF-Tx2 and Chloroquine were assessed for Gi signaling (f) and Gq signaling (g). The bias factor (β value) of CNF-Tx2 and Chloroquine was calculated using BAM8-22 as the reference.

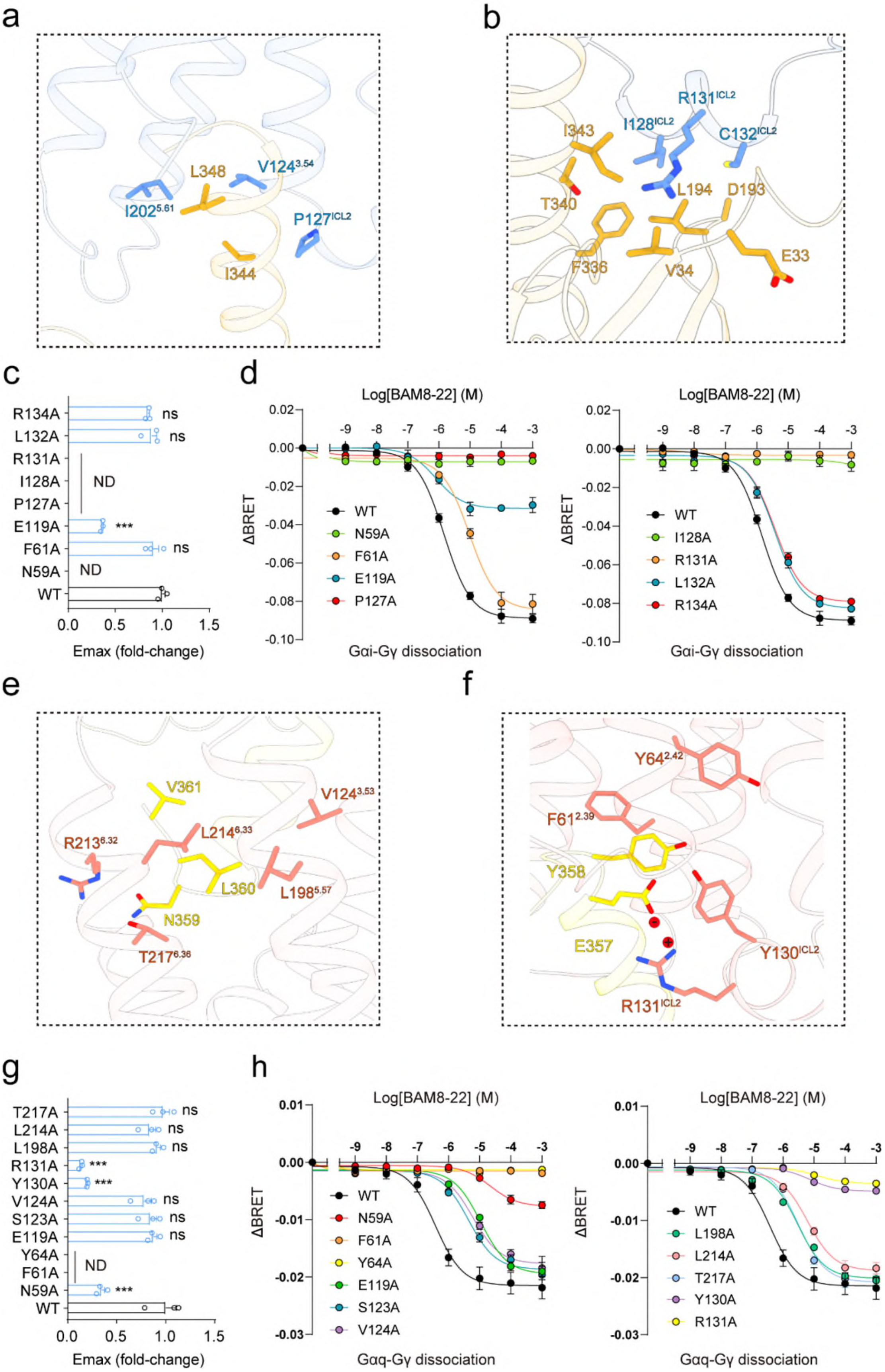
a
L348
V124 3.54
I202 5.61
P127 ICL2
I344
b
R131 ICL2
I128 ICL2
C132 ICL2
I343
T340
L194
D193
F336
V34
E33
c
R134A ns
L132A ns
R131A
I128A ND
P127A
E119A ***
F61A ns
N59A ND
WT
0.0 0.5 1.0 1.5
Emax (fold-change)
d
Log[BAM8-22] (M)
ΔBRET
WT
N59A
F61A
E119A
P127A
Gαi-Gγ dissociation
Log[BAM8-22] (M)
WT
I128A
R131A
L132A
R134A
Gαi-Gγ dissociation
e
V361
V124 3.53
L214 6.33
R213 6.32
L360
L198 5.57
N359
T217 6.36
f
Y64 2.42
F61 2.39
Y358
E357
Y130 ICL2
R131 ICL2
g
T217A ns
L214A ns
L198A ns
R131A ***
Y130A ***
V124A ns
S123A ns
E119A ns
Y64A
F61A ND
N59A ***
WT
0.0 0.5 1.0 1.5
Emax (fold-change)
h
Log[BAM8-22] (M)
ΔBRET
WT
N59A
F61A
Y64A
E119A
S123A
V124A
Gαq-Gγ dissociation
Log[BAM8-22] (M)
WT
L198A
L214A
T217A
Y130A
R131A
Gαq-Gγ dissociation

Supplementary Fig. 12 Coupling of MRPGRX1 with Gi and Gq.

(a) Detailed interactions between the TM bundles of MRGPRX1 and the α5-helix end of Gαi. **(b)** Detailed interactions between the ICL2 of MRGPRX1 and the Gαi. **(c)** Emax effects of different mutations of G protein interface mutations of MRGPRX1 on Gαi. Statistical differences between MRGPRX1 WT and mutations were presented as the mean ± SEM of three independent experiments and determined by two-sided one-way ANOVA with Tukey test. *, P<0.05; **, P<0.01; ***, P<0.001; ns, no significant difference; ND, not detected. (P=<0.001, <0.001, ND, ND, ND, <0.001, <0.001, ND from top to bottom). **(d)** The effects of G protein interface mutations of MRGPRX1 on Gαi. The curve data from three independent measurements are measured as mean ± SEM (n=3). **(e)** Detailed interactions between the bulky end of α5 helix of Gαq and the V124$^{3.54}$, L198$^{5.57}$, R213$^{6.32}$, L214$^{6.33}$ and T217$^{6.36}$ of MRGPRX1. **(f)** Detailed interactions between the E357$^{G.H5.22}$, Y358$^{G.H5.23}$ of Gαq and Y64$^{2.42}$, F61$^{2.39}$ and Y130^{ICL2}, R131^{ICL2} of MRGPRX1. **(g)** Emax effects of different mutations of G protein interface mutations of MRGPRX1 on Gαq. Statistical differences between MRGPRX1 WT and mutations were presented as the mean ± SEM of three independent experiments and determined by two-sided one-way ANOVA with Tukey test. *, P<0.05; **, P<0.01; ***, P<0.001; ns, no significant difference; ND, not detected. (P=0.0007, <0.001, 0.0222, <0.001, <0.001, <0.001, 0.0005, <0.001, ND, ND, <0.001 from top to bottom). **(h)** The effects of G protein interface mutations of MRGPRX1 on Gαq. The curve data from three independent measurements are measured as mean ± SEM (n=3).

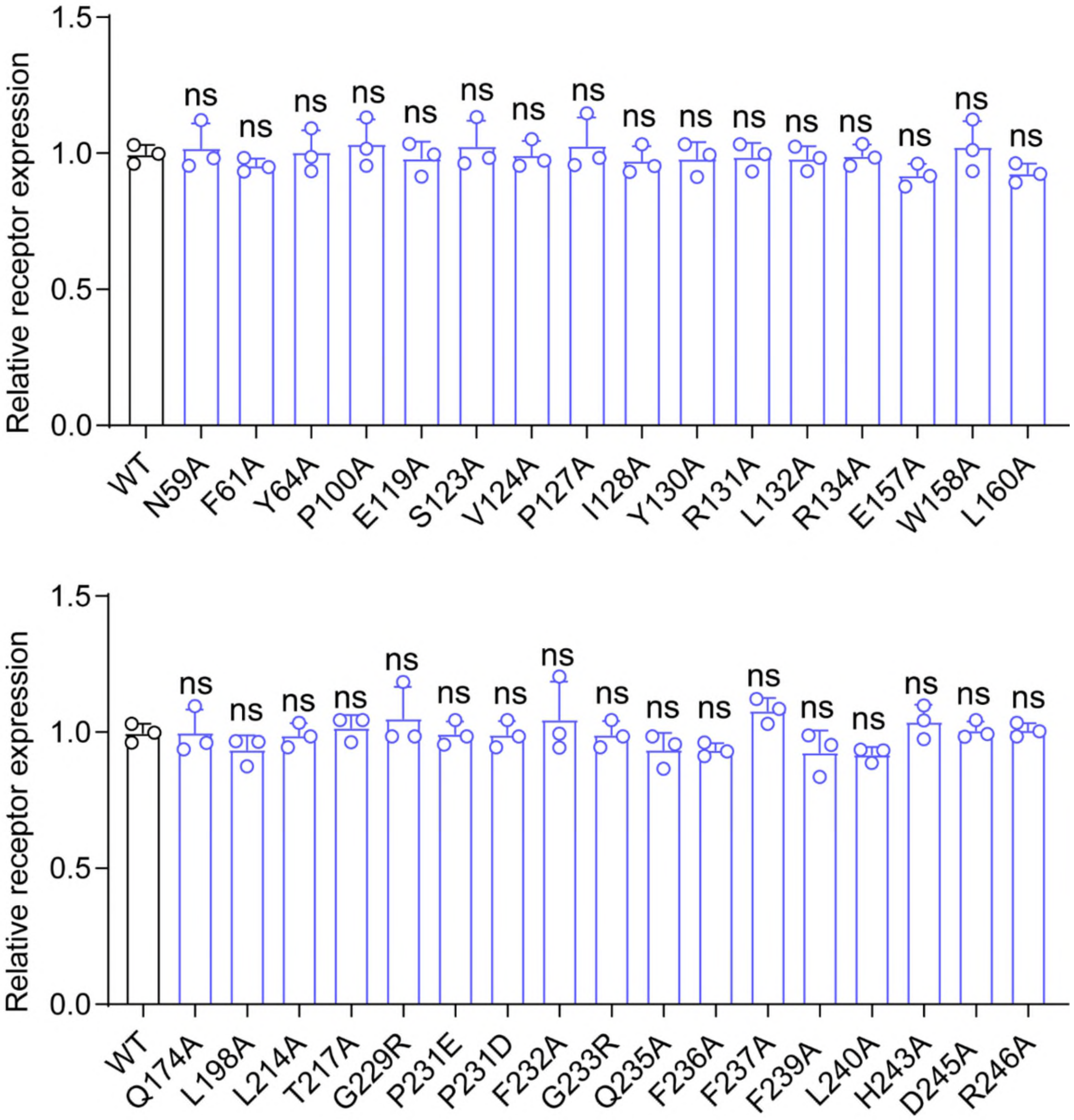

Supplementary Fig. 13 Expression level of relative MRGPRX1 wild type or mutants determined by ELISA.

Values are mean ± SEM from three independent experiments (n=3) performed in triplicates. ns, no significance. All data were analyzed by two-sided one-way ANOVA with Turkey test.

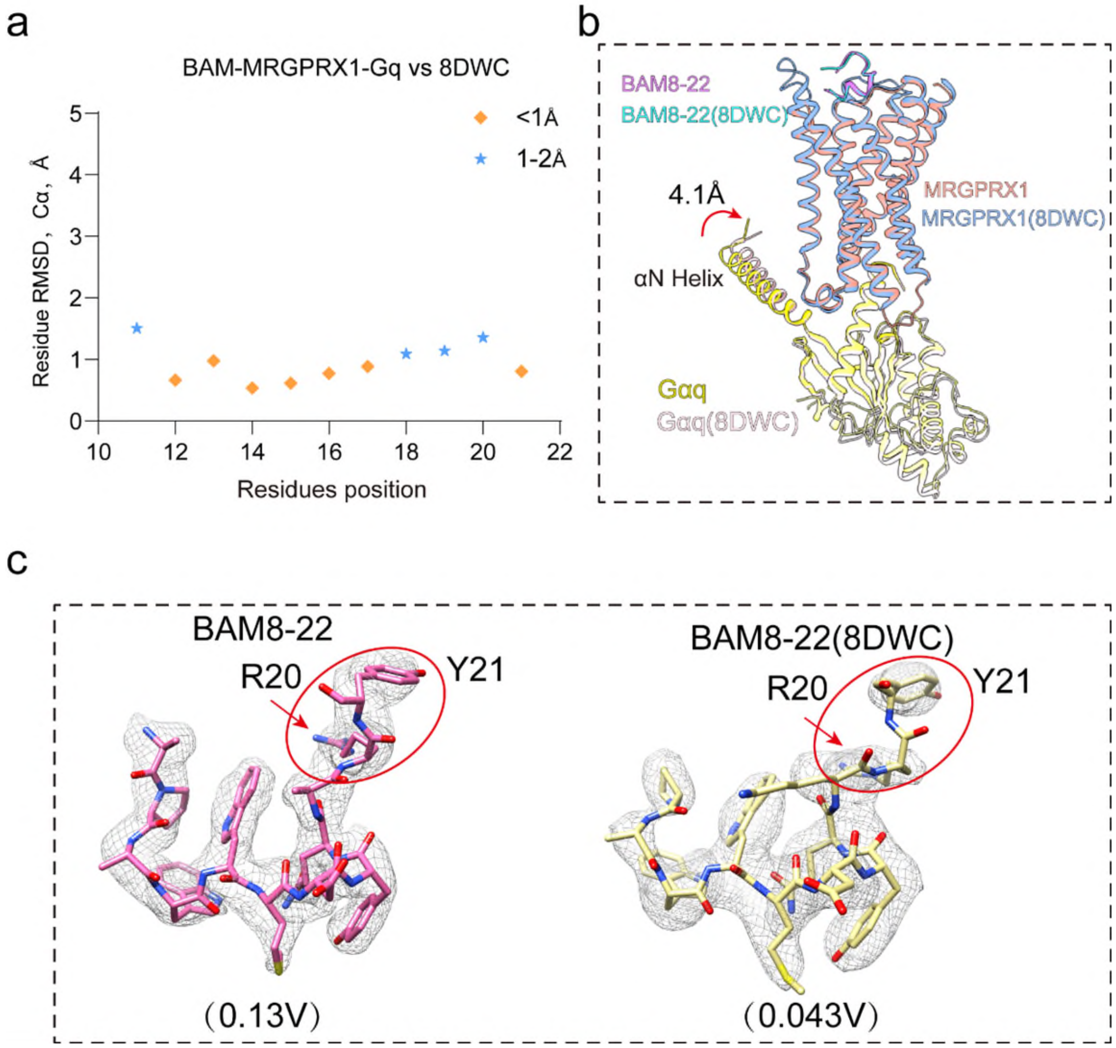

Supplementary Fig. 14 Comparison of the structure of BAM8-22-MRGPRX1-Gq complex (we solved vs. PDB ID: 8DWC).

(**a**) The root-mean-square-deviation (RMSD) of BAM8-22 between BAM8-22-MRGPRX1-Gq and 8DWC complex structures. **(b)** Comparison of the difference in MRGPRX1 TMs and G protein between the newly solved BAM8-22-MRGPRX1-Gq structure (green) and the structure solved by Yongfeng Liu et al. (PDB: 8DWC) through a cytoplasmic view. **(c)** Comparison of ligand densities in the BAM8-22-MRGPRX1-Gq and 8DWC structures.

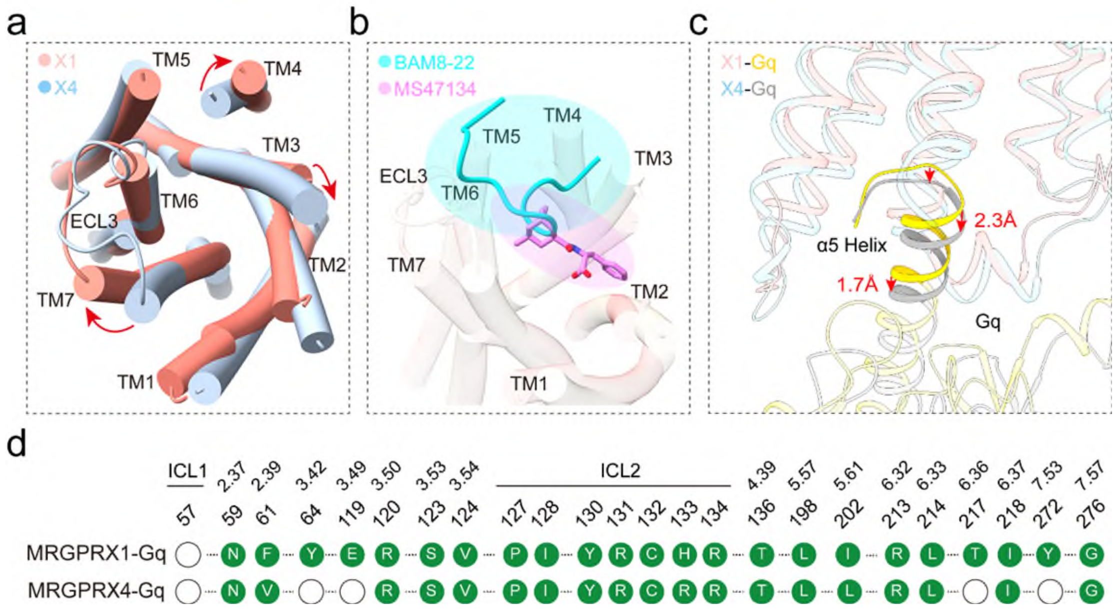

Supplementary Fig. 15 Structural comparison of MRGPRX1 with MRGPRX4.

(**a**) A cytoplasmic view of the BAM8-22-MRGPRX1 7TM bundle compared with MS47134-MRGPRX4 (PDB ID: 7S8P). MRGPRX1 is shown in salmon, MRGPRX4 in light sky blue. (**b**) Three-dimensional (3D) representation of BAM8-22 in the MRGPRX1 and MS47134-MRGPRX4 (PDB ID: 7S8P). BAM8-22 is shown in cyan, MS47134 in hot pink. (**c**) The structural representation and comparison of the interfaces between the MRGPRX1-Gq and MRGPRX4-Gq complexes. Ribbon representation: Gq bound to MRGPRX1 is shown in yellow, Gq bound to MRGPRX4 is shown in gray. (**d**) Comparison of the Gq coupling interfaces in cryo-EM structures of BAM8-22-MRGPRX1-Gq and MS47134-MRGPRX4 (PDB ID: 7S8P) complexes. Residues of MRGPRX1 in contact with Gq were illustrated as green dots.

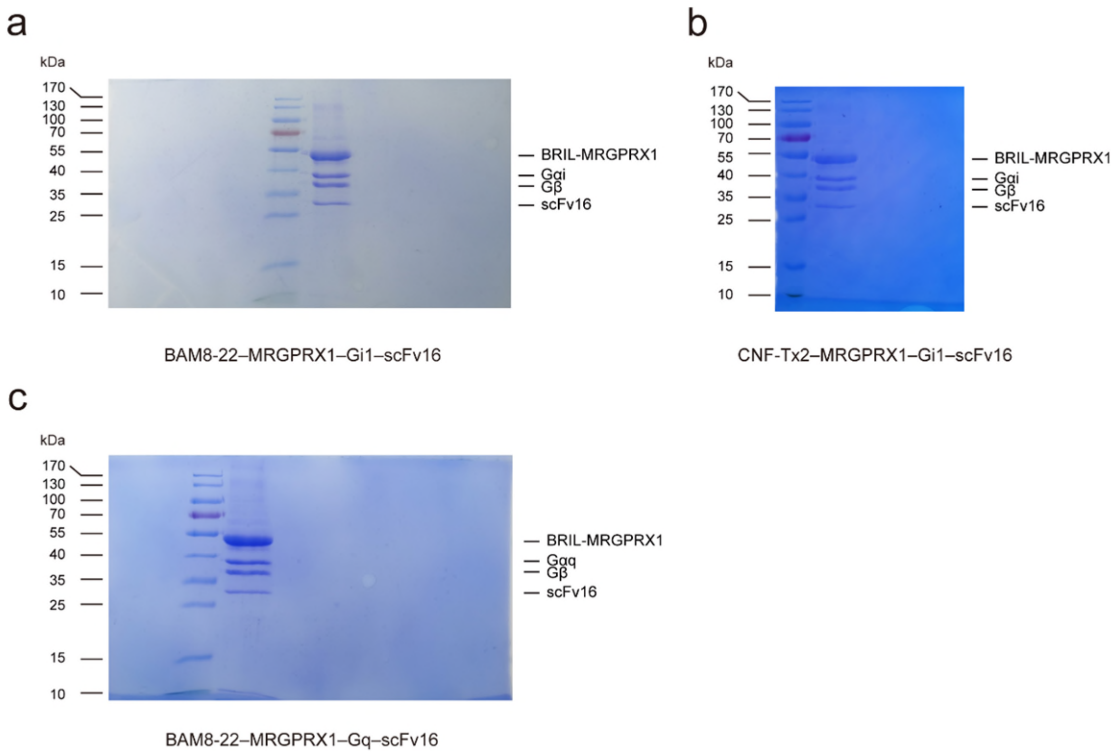

Supplementary Fig. 16 (a-c) SDS-PAGE of the size-exclusion chromatography peak of MRGPRX1-Gi1-scFv16 and MRGPRX1-Gq-scFv16 complex. (Raw data of Supplementary Fig. 1)

Supplementary Table 1: Cryo-EM Data Collection, Model Refinement, and Validation Statistics.

	BAM8-22-MRGPRX1-Gq (EMDB-36232) (PDB 8JGF)	CNF-Tx2-MRGPRX1-Gi (EMDB-36229) (PDB 8JGB)	BAM8-22-MRGPRX1-Gi (EMDB-36233) (PDB 8JGG)
**Data collection and processing**			
Magnification	81,000	130,000	130,000
Voltage (kV)	300	300	300
Electron exposure (e–/Å^2)	50	50	60
Defocus range (μm)	-1.2 to -2.2	-0.8 to -1.2	-0.8 to -1.2
Pixel size (Å)	1.04	1.08	0.89
Symmetry imposed	C1	C1	C1
Initial particle images (no.)	11,127,531	146,824,9	3,628,139
Final particle images (no.)	1,316,443	315,448	925,644
Map resolution (Å)	2.7	2.8	3.0
FSC threshold	0.143	0.143	0.143
Map resolution range (Å)	2.0-3.5	2.0-3.5	2.0-3.5
**Refinement**			
Initial model used (PDB code)	7UVY	7UVY	7UVY
Model resolution (Å)	3.1	3.0	3.2
FSC threshold	0.5	0.5	0.5
Model resolution range (Å)	2.0-3.5	2.0-3.5	2.0-3.5
Map sharpening *B* factor (Å^2)	-129.7	-101.8	-137.2
Model composition			
Non-hydrogen atoms	8338	7807	7889
Protein residues	1101	1062	1076
Ligands	1	1	1
B factors (Å^2)			
Protein	27.93	50.79	92.83
R.m.s. deviations			
Bond lengths (Å)	0.008	0.010	0.007
Bond angles (°)	1.105	1.384	1.145
Validation			
MolProbity score	1.78	2.16	1.92
Clashscore	8	7	9
Poor rotamers (%)	0.1	0	0.7
Ramachandran plot			
Favored (%)	95.74	93.82	94.30
Allowed (%)	4.26	6.18	5.70
Disallowed (%)	0	0	0

Supplementary Table 2: The summary of models and maps. Related to Figure 1 to Figure 5.

Model	Map	Contour Level	Residue	Description
BAM8-22_MRGPRX1_Gi_model	BAM8-22_MRGPRX1_Gi_map	3.60 rmsd	Receptor: L28-G276<br>Gαi: S6-I55, T182-F354<br>Gβ: D5-N340<br>Gγ: S8-K64<br>scFv: V2-L235<br>ECL1, ECL2 and ICL3 are in general disordered in GPCR structures and are not modeled.	(1) In the section of "Overall structures of MRGPRX1 complexes", used for structural descriptions for BAM8-22_MRGPRX1_Gi complex (Fig. 1 and Supplementary Fig.5)<br>(2) In the section of "Binding of BAM8-22 to MRGPRX1" (Fig. 2 and Supplementary Fig.7)<br>(3) In the section of "Coupling of MRPGRX1 with Gi and Gq" (Fig. 5 and Supplementary Fig. 10)
BAM8-22_MRGPRX1_Gq_model	BAM8-22_MRGPRX1_Gq_map	4.10 rmsd	Receptor: Y24-R279<br>Gαq: A7-R54, T182-V361<br>Gβ: D10-N345<br>Gγ: A9-R61<br>scFv: V1-L247<br>ECL1, ECL2 and ICL3 are in general disordered in GPCR structures and are not modeled.	(1) In the section of "Overall structures of MRGPRX1 complexes", used for structural descriptions for BAM8-22_MRGPRX1_Gq(Fig. 1 and Supplementary Fig.5)<br>(2) In the section of "Binding of BAM8-22 to MRGPRX1" (Fig. 2 and Supplementary Fig.7)<br>(3) In the section of "Coupling of MRPGRX1 with Gi and Gq" (Fig. 5 and Supplementary Fig. 10)
CNF-Tx2_MRGPRX1_Gi_model	CNF-Tx2_MRGPRX1_Gi_map	0.95 rmsd	Receptor: L28-F278<br>Gαi: L5-I55, T182-L353<br>Gβ: D5-N340<br>Gγ: S8-K64<br>scFv: V2-L235<br>ECL1, ECL2 and ICL3 are in general disordered in GPCR	(1) In the section of "Overall structures of MRGPRX1 complexes", used for structural descriptions for CNF-Tx2_MRGPRX1_Gi (Fig. 1 and Supplementary Fig. 5)<br>(2) In the section of "Binding of CNF-Tx2 to MRGPRX1", used for structural descriptions for CNF-Tx2_MRGPRX1_Gi complex (Fig. 3)

			structures and are not modeled.	

Supplementary Table 3. The residues with ambiguous side chain densities that only main chain atoms kept in the model of BAM8-22-MRGPRX1-Gi complex, BAM-MRGPRX1-Gq complex and CNF-Tx2-MRGPRX1-Gi complex.

	BAM8-22-MRGPRX1-Gi complex	BAM8-22-MRGPRX1-Gq complex	CNF-Tx2-MRGPRX1-Gi complex
Ligand	E12, D16, K19	R10, E12, K19	R14, R17
TM1	L28, L30, V32, L33, L39, W50, L52, C54, R55	Q26, L28, S29, V32, L33, T34, C35, S38, L49	L30, L33, T31, T34, L39, W50, L51
ICL1	R57	R55, M56, R57, R58, N59	-/-
TM2	L80, S86, F87	D72, I81	L66, D72, F73, R79, L80, Y82
TM3	I97	S114	-/-
TM4	-/-	S139, H137, M159	H137, C161
TM5	T172, Q174	Q174, F178, R177, V190, C204	S176, F178, T180, C204
ICL3	-/-	S206, R207, K208, I209	-/-
TM6	T212, Y215, C228, F239, H243	L211, L219	T212, R213, Y215, L219, L220, L227
ECL3	E247, V248	E247	-/-
TM7	C251, H252, F259, S265, S266	-/-	V256, N264, F274

Supplementary Table 4. The residues with ambiguous main chain densities that not modelled in the BAM8-22-MRGPRX1-Gi complex, BAM8-22-MRGPRX1-Gq complex and CNF-Tx2-MRGPRX1-Gi complex.

	BAM8-22-MRGPRX1-Gi complex	BAM8-22-MRGPRX1-Gq complex	CNF-Tx2-MRGPRX1-Gi complex
N-terminal	Y24, K25	L22	-/-
TM1	Q26, T27	-/-	I36, L52, G53, C54, R55, M56
ICL1	-/-	-/-	R57, R58
TM2	I88	-/-	-/-
ECL1	S89	T93	-/-
TM3	I94, S95, K96	I94	-/-
TM4	L160	-/-	L151
ECL2	C162	F163, L164, F165, S166, G167, A168	-/-
TM5	-/-	D169, S170, A171	-/-
ICL3	K208, I209, L211	G205, I209	G205, S206, R207, K208, I209
TM6	P210	P210	P210
ECL3	D245, R246, E247, V248	-/-	-/-
TM7	S277, F278	-/-	L249, G276, S277, F278

Supplementary Table 5: Interaction of BAM8-22 and MRGPRX1 in BAM8-22-MRGPRX1-Gi complex.

Interaction	MRGPRX1	BAM8-22	Distance (Å)
Hydrogen bond (≤ 3.5 Å)	E157	Y21	2.6
	W241	W14	2.8
Hydrophobic and van der Waals force (≤ 4.5 Å)	P100	Y21	4.5
	E157	Y21	4.1
		R20	2.9
	W158	Y21	3.5
	F236	Y17	3.9
		R20	3.8
	F237	R20	3.2
	L240	W14	3.8
		W13	2.8
		Q18	4.0
	W241	W13	3.7
		P11	3.5
	I242	W13	3.8
	H243	W13	4.0
	R246	M15	4.0
	L249	M15	4.2
		Y17	3.4
		Q18	3.2
	F250	Y17	4.3
	H254	Y17	3.0
Polar (≤ 4.5 Å)	Y99	R20	3.6
	S154	Y21	3.5
	E157	R20	2.7
	D177	R20	3.4
π-π stacking (Edge-π, ≤ 8.0 Å)	Y99	Y21	3.7
	W158	Y21	3.5
	F239	W13	7.4
	F250	Y17	3.3

Supplementary Table 6: Interaction of BAM8-22 and MRGPRX1 in BAM8-22-MRGPRX1-Gq complex.

Interaction	MRGPRX1	BAM8-22	Distance (Å)
Hydrophobic and van der Waals force (≤ 4.5 Å)	Y99	Y21	3.5
Hydrophobic and van der Waals force (≤ 4.5 Å)	Y99	R20	3.2
Hydrophobic and van der Waals force (≤ 4.5 Å)	P100	Y21	3.8
Hydrophobic and van der Waals force (≤ 4.5 Å)	S154	Y21	4.4
Hydrophobic and van der Waals force (≤ 4.5 Å)	E157	Y21	3.8
Hydrophobic and van der Waals force (≤ 4.5 Å)	W158	Y21	3.7
Hydrophobic and van der Waals force (≤ 4.5 Å)	I242	W13	4.0
Hydrophobic and van der Waals force (≤ 4.5 Å)	H243	W13	3.4
Hydrophobic and van der Waals force (≤ 4.5 Å)	R246	M15	3.1
Hydrophobic and van der Waals force (≤ 4.5 Å)	L249	Y17	3.1
Hydrophobic and van der Waals force (≤ 4.5 Å)	L249	Q18	3.5
Hydrophobic and van der Waals force (≤ 4.5 Å)	F250	Y17	3.4
Hydrophobic and van der Waals force (≤ 4.5 Å)	H254	Y17	3.3
Hydrogen bonds (≤ 3.5 Å)	E157	R20	2.7
Hydrogen bonds (≤ 3.5 Å)	D177	R20	2.7
Hydrogen bonds (≤ 3.5 Å)	L240	W14	2.8
Polar (≤ 4.5 Å)	F239	Q18	3.9
π-π stacking (Edge-π, ≤ 8.0 Å)	Y82	Y17	6.9
π-π stacking (Edge-π, ≤ 8.0 Å)	W158	Y21	4.6
π-π stacking (Edge-π, ≤ 8.0 Å)	F236	Y17	4.3
π-π stacking (Edge-π, ≤ 8.0 Å)	F237	Y21	8.0
π-π stacking (Edge-π, ≤ 8.0 Å)	W241	R20	3.3
π-π stacking (Edge-π, ≤ 8.0 Å)	W241	W14	4.0

Supplementary Table 7: Interaction of CNF-Tx2 and MRGPRX1 in CNF-Tx2-MRGPRX1-Gi complex.

Interaction	MRGPRX1	CNF-Tx2	Distance (Å)
Hydrophobic and van der Waals force (≤ 4.5 Å)	S95	I18	4.2
	Y99	I18	3.3
	L240	R17	3.6
π-π stacking (Edge-π, ≤ 8.0 Å)	Y82	F15	7.4
	Y99	F15	7.0
	F236	F15	4.0

Supplementary Table 8: Interaction of CNF-Tx2 and MRGPRX1 in CNF-Tx2-MRGPRX1-Gi-MD complex.

Interaction	MRGPRX1	CNF-Tx2	Distance (Å)
Hydrophobic and van der Waals force (≤ 4.5 Å)	S95	I18	4.0
	Y99	I18	4.0
		R17	3.7
	L160	R14	4.4
		R17	3.6
	S176	R17	4.2
	F237	R17	4.0
	L240	R17	3.9
π-π stacking (Edge-π, ≤ 8.0 Å)	Y82	F15	7.0
	F236	F15	4.0
Hydrogen bond (≤ 3.5 Å)	E157	R17	2.8
	D177	R17	2.8

Supplementary Table 9: **Interactions between MRGPRX1 and Gαi in BAM8-22-MRGPRX1-Gi complex.**

Interactions	MRGPRX1	Gαi	Distance (Å)
Hydrophobic and van der Waals force (≤ 4.5 Å)	F61	D350	4.1
		C351	3.4
	R120	C351	3.8
	S123	N347	3.9
		C351	3.9
	V124	L348	4.4
	P127	I344	3.7
		N347	4.1
	I128	K192	4.4
		D193	4.0
		L194	3.6
		I343	4.3
	R131	R32	4.3
		E33	3.6
		V34	3.1
		I343	3.1
	C132	R32	4.0
		L194	3.9
	H133	L193	3.5
	R134	R32	3.6
	P135	R32	4.4
	T136	E28	3.6
	L198	L360	3.9
	I202	L348	4.3
	R213	L353	3.4
	R213	F354	3.8
	L214	L360	3.7
	T217	T340	4.0
	T217	I343	4.2
	I218	T340	4.2
Polar (≤ 4.5 Å)	N59	D350	4.3
	R120	C351	3.6
	S123	C351	3.9
	R131	R32	3.8

	R131	A31	4.2
		T219	4.0
	H133	R32	3.9
**Hydrogen bond (≤ 3.5 Å)**	S123	N347	3.1
	R131	E33	2.7
	C132	D193	3.2
	R134	R32	2.8
	T136	R32	2.8

Supplementary Table 10: **Interactions between MRGPRX1 and Gαq in BAM8-22-MRGPRX1-Gq complex.**

Interactions	MRGPRX1	Gαq	Distance (Å)
Hydrophobic and van der Waals force (≤ 4.5 Å)	N59	E357	4.4
	A60	E357	4.0
	F61	E357	3.6
		Y358	3.3
		N359	3.4
	V116	Y358	4.2
	E119	Y358	3.2
	R120	Y358	3.2
		L360	3.9
	I128	K347	3.9
		I350	3.8
	L198	L360	3.9
	I202	L355	4.5
	S123	N354	3.7
		Y358	3.3
	V124	L351	4.0
		L355	3.4
		L360	4.4
	P127	I350	3.1
		L351	3.7
	Y130	N354	3.5
		E357	3.3
		Y358	3.0
	R131	I350	3.3
		M353	4.0

	**R131**	**N354**	**3.6**
	**C132**	**R31**	**3.6**
		**R32**	**3.9**
	**H133**	**R32**	**3.5**
	**T136**	**K27**	**4.5**
	**P210**	**V361**	**4.1**
	**R213**	**L360**	**4.0**
		**N359**	**4.0**
		**V361**	**4.1**
	**L214**	**L355**	**4.3**
		**L360**	**3.4**
	**T217**	**N359**	**4.3**
	**I218**	**L360**	**4.1**
	**G276**	**N359**	**3.9**
**Hydrogen bond (≤ 3.5 Å)**	**P127**	**N354**	**3.3**
	**Y130**	**E357**	**2.4**
	**Y130**	**Y358**	**2.4**
	**R131**	**N354**	**2.6**
	**C132**	**R31**	**3.3**
	**R134**	**R31**	**2.9**
	**L214**	**L360**	**3.3**
	**Y272**	**N359**	**3.2**
**Polar (≤ 4.5 Å)**	**A60**	**E357**	**3.7**
	**H133**	**R32**	**3.7**
	**R213**	**N359**	**4.0**
	**T217**	**N359**	**3.7**
**π-π stacking (Edge-π, ≤ 8.0 Å)**	**Y64**	**Y358**	**5.9**
