# Supplementary material for: Ligand recognition and G protein coupling of the human itch receptor MRGPRX1
Source: Nat Commun. 2023 Aug 17;14:5004. doi: 10.1038/s41467-023-40705-z (PMC10435460; doi:10.1038/s41467-023-40705-z)
Supplement: Supplementary file 3 — Reporting Summary [file 41467_2023_40705_MOESM3_ESM.pdf]

nature portfolio

Corresponding author(s): Jin-peng Sun

Last updated by author(s): July 20,2023

Reporting Summary

Nature Portfolio wishes to improve the reproducibility of the work that we publish. This form provides structure for consistency and transparency in reporting. For further information on Nature Portfolio policies, see our Editorial Policies and the Editorial Policy Checklist.

Statistics

For all statistical analyses, confirm that the following items are present in the figure legend, table legend, main text, or Methods section.

n/a	Confirmed	
☐	☒	The exact sample size (*n*) for each experimental group/condition, given as a discrete number and unit of measurement
☐	☒	A statement on whether measurements were taken from distinct samples or whether the same sample was measured repeatedly
☐	☒	The statistical test(s) used AND whether they are one- or two-sided<br>*Only common tests should be described solely by name; describe more complex techniques in the Methods section.*
☒	☐	A description of all covariates tested
☒	☐	A description of any assumptions or corrections, such as tests of normality and adjustment for multiple comparisons
☐	☒	A full description of the statistical parameters including central tendency (e.g. means) or other basic estimates (e.g. regression coefficient) AND variation (e.g. standard deviation) or associated estimates of uncertainty (e.g. confidence intervals)
☐	☒	For null hypothesis testing, the test statistic (e.g. *F*, *t*, *r*) with confidence intervals, effect sizes, degrees of freedom and *P* value noted<br>*Give P values as exact values whenever suitable.*
☒	☐	For Bayesian analysis, information on the choice of priors and Markov chain Monte Carlo settings
☒	☐	For hierarchical and complex designs, identification of the appropriate level for tests and full reporting of outcomes
☒	☐	Estimates of effect sizes (e.g. Cohen's *d*, Pearson's *r*), indicating how they were calculated

Our web collection on statistics for biologists contains articles on many of the points above.

Software and code

Policy information about availability of computer code

Data collection	Automated data collection on the Titan Krios was performed using serialEM.
Data analysis	The following software was used in this study: MotionCor2.1, gCTF, RELION 4.0, UCSF Chimera, UCSF Chimera X, Coot, Phenix, Graphpad Prism, Pymol 2.1.

For manuscripts utilizing custom algorithms or software that are central to the research but not yet described in published literature, software must be made available to editors and reviewers. We strongly encourage code deposition in a community repository (e.g. GitHub). See the Nature Portfolio guidelines for submitting code & software for further information.

Data

Policy information about availability of data

All manuscripts must include a data availability statement. This statement should provide the following information, where applicable:

- Accession codes, unique identifiers, or web links for publicly available datasets
- A description of any restrictions on data availability
- For clinical datasets or third party data, please ensure that the statement adheres to our policy

The cryo-EM density map for the BAM8-22-MRGPRX1-Gq, CNF-Tx2-MRGPRX1-Gi and BAM8-22-MRGPRX1-Gi complexes have been deposited in the Electron Microscopy Data Bank (EMDB) under accession codes EMD-36232, EMD-36229, EMD-36233. The coordinates for the model of BAM8-22- MRGPRX1-Gq, CNF-Tx2-MRGPRX1-Gi and BAM8-22- MRGPRX1-Gi complexes have been deposited in the PDB under accession numbers 8JGF, 8JGB, and 8JGG. All other data are available

upon request to the corresponding authors.

Research involving human participants, their data, or biological material

Policy information about studies with human participants or human data. See also policy information about sex, gender (identity/presentation), and sexual orientation and race, ethnicity and racism.

Reporting on sex and gender	N/A
Reporting on race, ethnicity, or other socially relevant groupings	N/A
Population characteristics	N/A
Recruitment	N/A
Ethics oversight	N/A

Note that full information on the approval of the study protocol must also be provided in the manuscript.

Field-specific reporting

Please select the one below that is the best fit for your research. If you are not sure, read the appropriate sections before making your selection.

☒ Life sciences ☐ Behavioural & social sciences ☐ Ecological, evolutionary & environmental sciences

For a reference copy of the document with all sections, see nature.com/documents/nr-reporting-summary-flat.pdf

Life sciences study design

All studies must disclose on these points even when the disclosure is negative.

Sample size	For structural determination, 5,601 movies of BAM8-22-MRGPRX1-Gq complex, 3,085 movies of CNF-Tx2-MRGPRX1-Gi complex, 5,540 movies of BAM8-22-MRGPRX1-Gi complex were collected using an Titan Krios equipped with a Gatan K2 or K3 Summit direct electron detector. For ligand-binding, , Gαi- Gγ dissociation, Gαq- Gγ dissociation and Elisa assays, at least three biologically independent experiments (n=3) were performed as depicted in related Figure legends. Data were analyzed by fitting various ligand concentrations and readouts using appropriate equations in GraphPad Prism 8.0.
Data exclusions	No data was systematically excluded. The procedure of generating 3D maps from cryo-EM particles involves sorting of particles that are damaged or are false-picked that are unlikely to refine correctly. This is implemented in RELION 4.0.
Replication	Each experiment was reproduced at least three times on separate occasions. Experimental findings were reliably reproduced.
Randomization	No randomization was attempted or needed. Randomization was not necessary as the independent variables to be tested were sufficient for the functional interpretation within this study. i.e. WT vs mutant vs control conditions or dose-response determination.
Blinding	Blinding is not necessary or valid for the purposes of structural determination. For cryo-EM study, purified ligand bound-MRGPRX1-Gα complex samples were applied onto a glow-discharged holey carbon grid and subsequently vitrified using a Vitrobot Mark IV. cryo-EM imaging was collected on a Titan Krios equipped with a Gatan K2 or K3 Summit direct electron detector. The microscope was operated at 300 kV accelerating voltage, at a nominal magnification of x130,000 in counting mode, with pixel sizes on the object scale being 1.04 Å(BAM8-22-MRGPRX1-Gq), 1.08 Å(CNF-Tx2- MRGPRX1-Gi) and 0.89 Å(BAM8-22- MRGPRX1-Gi). In total, 5,601 movies of BAM8-22-MRGPRX1-Gq complex, 3,085 movies of CNF-Tx2-MRGPRX1-Gi complex, 5,540 movies of BAM8-22-MRGPRX1-Gi were collected at an accumulated dose of 50, 50, 60 electrons per Å2 and a total of 36, 32, 32 frames per movie, respectively. The defocus values ranged from -0.8 to -2.2um. For functional analysis, blinding was not necessary due to the quantitative nature of the experiment. All experimental data acquired or analyzed in this study are included in this published article, and subjected to statistical analysis whenever necessary.

Reporting for specific materials, systems and methods

We require information from authors about some types of materials, experimental systems and methods used in many studies. Here, indicate whether each material, system or method listed is relevant to your study. If you are not sure if a list item applies to your research, read the appropriate section before selecting a response.

Materials & experimental systems

n/a	Involved in the study
☐	☒ Antibodies
☐	☒ Eukaryotic cell lines
☒	☐ Palaeontology and archaeology
☒	☐ Animals and other organisms
☒	☐ Clinical data
☒	☐ Dual use research of concern
☒	☐ Plants

Methods

n/a	Involved in the study
☒	☐ ChIP-seq
☒	☐ Flow cytometry
☒	☐ MRI-based neuroimaging

Antibodies

Antibodies used

For measurement of receptor cell surface expression, monoclonal anti-FLAG primary antibody (Sigma Aldrich, Catalog # F1804) and goat anti-mouse secondary antibody (Thermo Fisher, Catalog # 31430) were used. The primary antibody was used in 1:1000 dilution, and the secondary antibody in 1:5000 dilution.

Validation

All antibodies used are commercially purchased and have been validated by the vendors. All antibodies are well characterized and were applied according to data sheet information details.
Monoclonal anti-FLAG: https://www.sigmaaldrich.com/catalog/product/sigma/f1804;
Goat anti-Mouse secondary antibody: https://www.thermofisher.com/cn/zh/antibody/product/Goat-anti-Mouse-IgG-H-LCrossAdsorbed-Secondary-Antibody-Polyclonal/A-21235.

Eukaryotic cell lines

Policy information about cell lines and Sex and Gender in Research

Cell line source(s)

HEK293 cells were obtained from Cell Resource Center of Shanghai Institute for Biological Sciences (Chinese Academy of Sciences, Shanghai, China). Sf9 cells were purchased from Expression Systems (Cat 94-001S).

Authentication

All of the cell lines are maintained by the supplier. No additional authentication was performed by the authors of this study.

Mycoplasma contamination

Cell lines are tested by manufacturer for contamination. Sf9 and HEK293 cell lines tested negative for mycoplasma contamination has been declared in methods section.

Commonly misidentified lines
(See ICLAC register)

No commonly misidentified cell lines were used.
